# Biodegradation and Utilization of Organophosphorus Pesticide Malathion by Cyanobacteria

**DOI:** 10.1155/2014/392682

**Published:** 2014-04-17

**Authors:** Wael M. Ibrahim, Mohamed A. Karam, Reda M. El-Shahat, Asmaa A. Adway

**Affiliations:** ^1^Botany Department, Faculty of Science, Fayoum University, Fayoum, Egypt; ^2^Agricultural Research Center, Ministry of Agriculture, Egypt

## Abstract

Three strains of filamentous Cyanobacteria were used to study their growth and utilization of organophosphorus pesticide malathion. A sharp decrease in the growth of the algal strains was observed by increasing the concentration of malathion. Amongst them *Nostoc muscorum* tolerated different concentrations and was recorded as the highest efficient strain for biodegradation (91%) of this compound. Moreover, carbohydrate and protein content of their cells overtopped the other strains especially at higher concentrations. The algal strains were further subjected to grow under P-limitation in absence and presence of malathion. Although, the algal growth under P-limitation recorded a very poor level, a massive enhanced growth and phosphorous content of cells were obtained when the P-limited medium was amended with malathion. This study clarified that *N. muscorum* with its capability to utilize malathion as a sole phosphorous source is considered as an inexpensive and efficient biotechnology for remediation of organophosphorus pesticide from contaminated wastewater.

## 1. Introduction

As a result of human impact, the levels of organic compounds found in surface water have increased in the recent decades. Of these organic compounds, pesticides are most commonly detected in all aquatic environments [1]. These pesticides are mainly used for agricultural purposes [2]. They enter the aquatic environment via runoff after being sprayed in agricultural fields and can potentially reach groundwater [3].

Malathion is a nonsystemic, wide spectrum organophosphate pesticide (OP), used to control insects on field crops, fruits, vegetables and also extensively used to prevent mosquitoes, flies, household insects, animal parasites, and head body lice [4].

Recent research shows that malathion has a variety of syndromes and effects including hepatotoxicity [5–7], human breast carcinoma [8], genetic damage [9], and disrupted normal hormone activity [10].

Not only are the chemical and physical methods of decontamination expensive and time-consuming, but also in most cases they do not provide a complete solution. Bioremediation provides a suitable way to remove contaminants from the environment as, in most of the cases, OP compounds are totally mineralized by the microorganisms. Most OP compounds are degraded by microorganisms in the environment as a source of phosphorus or carbon or both [11].

Photoautotrophic microorganisms, such as Cyanobacteria, are used for wastewater treatment to remove nitrogen and phosphorus [12]. They have potential to remove various pollutants, such as dyes [13], heavy metals [14], and pesticides [15]. Therefore, this study is conducted to investigate the survival and tolerance of cyanobacterial isolates* Anabaena oryzae*,* Nostoc muscorum,* and* Spirulina platensis* with different concentrations of malathion, as well as evaluating their efficiency for removing and recovering this pesticide from contaminated wastewater.

## 2. Material and Methods

### 2.1. Algal Strains

The algal strains (*Anabaena oryzae *and* Nostoc muscorum*) were isolated from different water samples collected from Al-Fayoum Governorate, Egypt. Whereas,* Spirulina platensis* was obtained from Agricultural Research Center, Ministry of Agriculture, Giza, Egypt.

### 2.2. Chemicals

The organophosphorus pesticide used in this study is commercially available as Malathion, chemical name (O,O-dimethyl-S-[1,2-di(ethoxycarbonyl)ethyl]phosphorodithioate) was obtained from Kafr Elzayyat company, Egypt (98% active ingredient).

### 2.3. Experimental Design

The selected algal isolates were batch-cultured in 500 mL Erlenmeyer flasks. Into each flask 200 mL of liquid culture media, BG11 medium [16] for* A. oryzae *and* N. muscorum* and Zarrouk medium [17] for* S. platensis, *was added. The initial inoculum was approximately 5 × 10^4^ cell/mL. Malathion was added to the culture medium to the final concentrations 0.02, 0.2, 2, 20, 50, or 100 ppm. The culture flasks were kept under continuous illumination provided by daylight fluorescent tubes with an average light intensity of 40 *μ*Em^−2^ s^−1^ maintained constantly during the experiment. The flasks were incubated in a culture room at 28 ± 1°C under continuous shaking of 80 rpm. Samples were taken after every four-day intervals up to fifty-two days for the estimation of the growth in terms of cell count. After 20 days, 50 mL of algal cultures was filtrated by centrifugation at 1500 rpm for 20 minutes. The algal filtrate was used to determine malathion residues in the culture medium.

To obtain phosphorus-limited cultures, exponentially growing cells were inoculated into flasks containing medium with 1/10th of the original phosphorus concentration. The phosphorus-limited cells were cultured in a medium without and with different concentrations of malathion. Samples were taken after 20 days for estimation of cell count and phosphorus content in the tested algal cells.

### 2.4. Analytical Analysis

Different algal cultures were sonicated with Ultrasonic Homogenizer (Model: cp100, USA) to make them short fragments; 10 mL of algal solution was placed on vials containing 0.1 mL Lugol's solution [18]. Cell count was carried out using a standard haemocytometer under an Olympus BH-2 light microscope. Protein content of algal biomass was determined according to Lowry et al. [19]. For the determination of carbohydrate content in algal cells, the anthrone sulphuric acid method which was carried out by Fales [20] and adopted by Irigoyen et al. [21] was used. The total phosphorus content in the algal biomass was measured spectrophotometricallo at 720 nm according to Pierpoint [22]. Hewlett Packard Agilent GC System (Gas Chromatograph, USA) Model 6890 equipped with a flame photometric detector (FPD) with phosphorus filter was used for determination of malathion residues in the culture medium.

### 2.5. Statistical Analysis

Data were presented as mean of replicates from three runs and were analyzed statistically using Student's *t*-test for independent samples. Statements of significant differences were based on accepting *P* ≤ 0.05. To validate the tolerance of algal strains, two identical series of linear regression curves were established for growth experiment.

## 3. Results

### 3.1. Effect of Malathion on Growth of Tested Algal Strains

**Figure 1:**
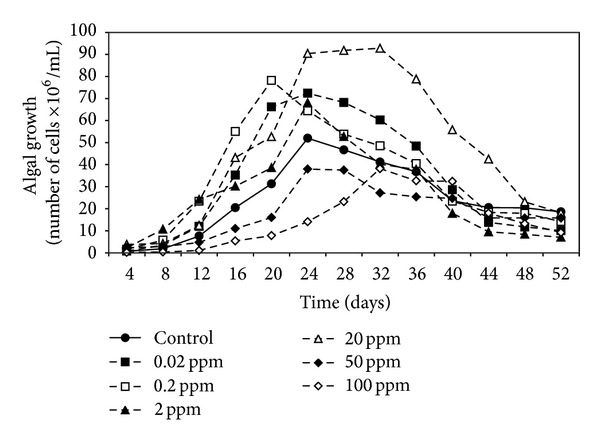
Effect of different malathion concentrations on the growth of* A. oryzae.*

**Figure 2:**
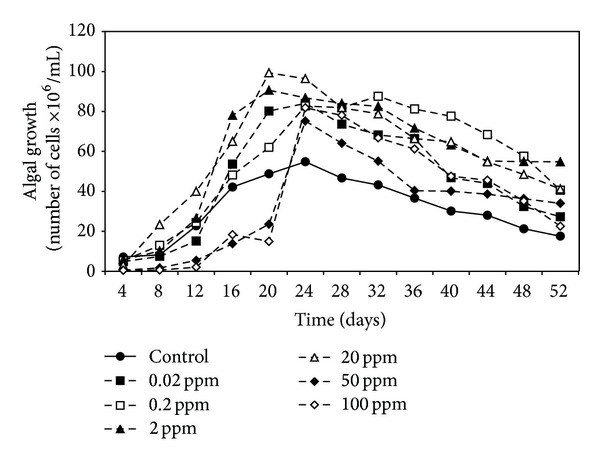
Effect of different malathion concentrations on the growth of* N. muscorum.*

**Figure 3:**
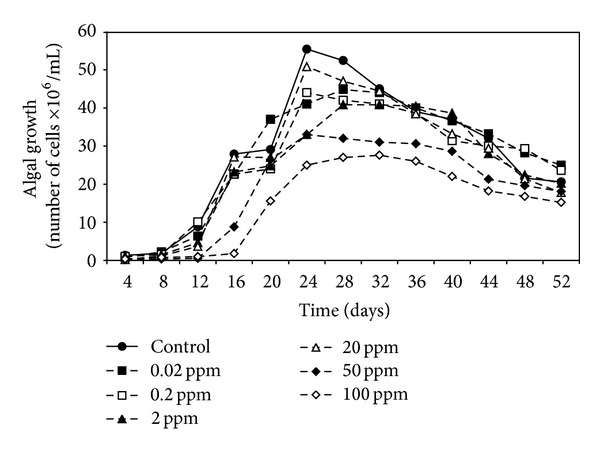
Effect of different malathion concentrations on the growth of* S. platensis.*

**Figure 4:**
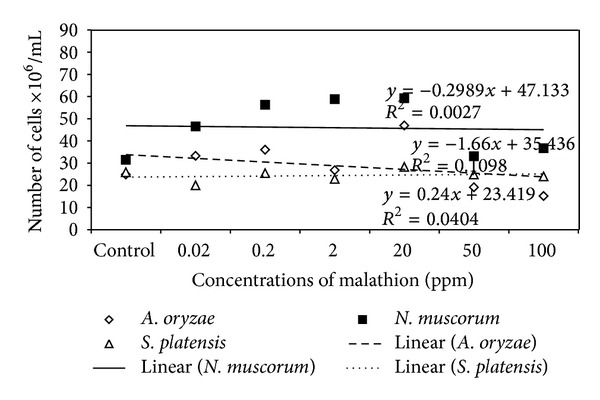
Regression lines of algal growth (expressed as cell count) of tested strains.

Data in Figures 1, 2, and 3 demonstrated the effect of malathion concentrations on the growth of three cyanobacterial strains,* A. oryzae*,* N. muscorum, *and* S. platensis*. Obviously, an inverse relationship between malathion concentration and the algal growth was recorded. At low concentrations of malathion (0.02–20 ppm), the maximum growth of* A. oryzae* and* N. muscorum *was achieved within 24 days recording an increment of the total cell number by 41% and 75%, respectively, compared with the untreated culture. At the same time, different malathion concentrations dramatically reduced the growth of* S. platensis *recording a reduction of the total cell count by 19% compared with the control treatment. Regression lines (Figure 4) indicated that* N. muscorum *was more tolerant than the other algal strains with different concentrations of malathion.

### 3.2. Effect of Malathion on Carbohydrate and Protein Content of Algal Cells

**Figure 5:**
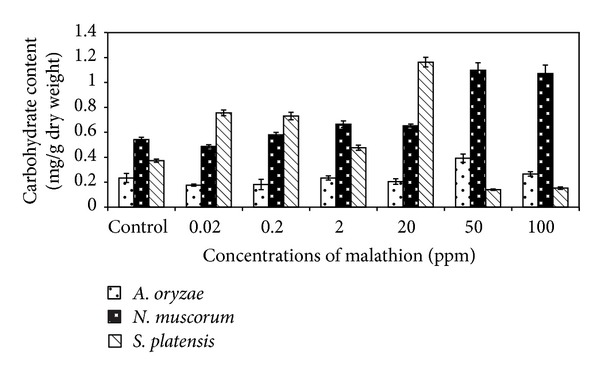
Effect of different concentrations of malathion on carbohydrate content of algal biomass. Data are the means of three replicates and error bars represent the standard errors of the means.

Data present in Figure 5 indicated that the treatment of* A. oryzae* and* N. muscorum* with different malathion concentrations caused a very high significant increase in total carbohydrate content with increasing concentrations of malathion and the highest carbohydrate content (0.39 and 1.09 mg/g dry weight, resp.) was recorded at 50 ppm of malathion. At the same time, carbohydrate content of* S. platensis* was increased until 20 ppm of malathion and then dramatically decreased as malathion concentration increased further.

**Figure 6:**
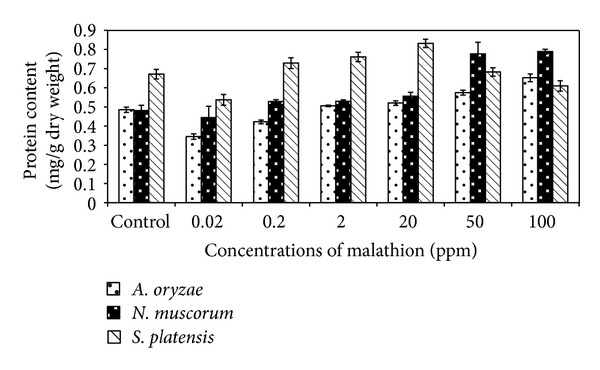
Effect of different concentrations of malathion on protein content of algal biomass. Data are the means of three replicates and error bars represent the standard errors of the means.

Concerning protein content of algal strains, it is clear from Figure 6 that treatment of* A. oryzae* and* N. muscorum* with malathion significantly increased protein content of cells especially at higher concentrations (50 and 100 ppm). In case of* S*.* platensis*, lower concentrations of malathion (0.2 and 20 ppm) caused significant increase in protein content of algal cells and high concentrations caused gradual decrease in protein content.

### 3.3. Biodegradation of Malathion by Different Algal Strains

**Figure 7:**
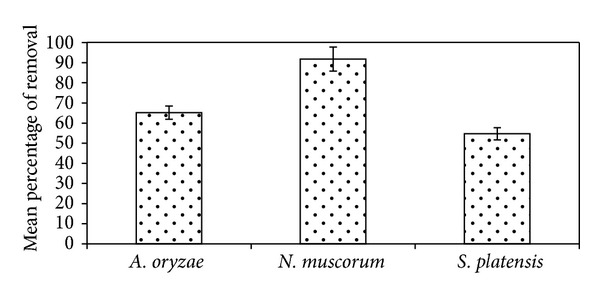
Efficiency of different algal strains to biodegrade malathion. Error bars represent the standard errors of the means.

Data present in Figure 7 illustrated that the three algal strains have the ability to biodegrade malathion at different concentrations. In general,* N. muscorum* was recorded as the highest efficient strain followed by* A. oryzae* and the lowest one was* S. platensis* with mean removal values of 91%, 65%, and 54%, respectively.

### 3.4. The Ability of Algal Strains to Utilise Malathion as Phosphorus Source

Algal strains were grown under phosphorus limitation condition in absence and presence of malathion in order to investigate their ability to utilise malathion as a sole phosphorus source. In absence of malathion, the growth of cells was markedly dwindled under phosphorus limitation recording a decrement in the total cell count by 75.5% compared with the unlimited cells (Table 1). On the other hand, when malathion was used as a sole phosphate source for the growth of algal strains under P-limitation condition, the greatest cell number was achieved recording 39%, 52%, and 20% increase more than the same conditions without the addition of malathion for* A. oryzae*,* N. muscorum,* and* S. platensis*, respectively.

The ability of algal strains to use malathion as phosphate source was confirmed by analysing the internal phosphorus content inside the algal biomass. Data in Table 2 revealed that the total phosphorus content of the cells that were cultured in media with P-limitation was very minor. When the limited culture was amended with malathion, the amounts of total phosphorus were increased to the same range spotted in the unlimited cells.

## 4. Discussion

It is clear from the results that the growth of algal strains was decreased as malathion concentration increased. This inverse correlation between malathion concentration and the algal growth agrees with Ibrahim and Essa [15] and Ghadai et al. [23] who studied the effect of different concentrations (1–400 ppm) of organophosphorus pesticides on the growth of seven cyanobacterial strains. They found that the low concentrations stimulated the growth in terms of cell number and the higher concentrations dramatically reduced the algal growth. In this respect, extensive studies have been made concerning the inhibitory effects of organophosphorus pesticides on the cell count of different algal species [24–27].

The inhibitory effect of malathion could be attributed to the adsorption of this compound on the rich-lipid plasma membranes of the algal cells, thus, altering the membranes permeability [28] and diminishing photosynthetic activity [29, 30] as well as increasing reactive oxygen species (ROS) during stress [25].

Figure 4 indicated that* N. muscorum *was more tolerant to different concentrations of malathion than the other algal strains. In agreement with these results Nayak et al. [27] reported that* Nostoc* sp. tolerated more than* Anabaena *sp. to organophosphorus pesticide monocrotophos and can grow up to 150 ppm. Also, Kumar et al. [31] study the tolerance of three cyanobacterial strains to endosulfan and record the tolerance in the order of* N. muscorum* >* A. variabilis* >* A. fertilissima. *Highest tolerance of* N. muscorum *could be as a result of its highest ability to biodegrade malathion (91%) at different concentrations (Figure 7).

In general, data obtained from Figures 5 and 6 indicated that the total carbohydrate and protein content of algal biomass increased significantly with increasing malathion concentrations. Such a phenomenon may be due to the presence of some enzymes which can hydrolyse this organophosphorus compound and utilize malathion as nutrient sources [15, 32]. Ghadai et al. [23] found that the organophosphorus pesticide diazinon stimulates carbohydrate content of blue green alga,* A. cylindrica*.

When algal strains were cultured in P-limited medium supplemented with different malathion concentrations, a highly significant growth was obtained compared with the cells that were grown under the same conditions without malathion addition which recorded a sharp reduction in their growth. In accordance with such results, Ibrahim and Essa [15] studied the effect of malathion on the growth of* A. oryzae* under phosphorus limited conditions. They found that the growth of* A. oryzae* under P-limitation recorded a very poor level and a massive enhanced growth was obtained when the P-limited medium was amended with malathion. The simulative effect of malathion on growth could be as a result of the increment of the available phosphorus, resulting from degradation of this compound by algal strains. Therefore, total phosphorus content of algal biomass was estimated in order to confirm their capability to utilise malathion as a phosphorus source. Results in Table 2 illustrated that the phosphorus content of the cells which grew under P-limitation and in presence of malathion was much higher than that found in cells cultured under the same conditions but without the addition of malathion revealing the capability of this strain to break down and utilize malathion as a sole phosphorus source. These findings agreed with Subramanian et al. [33] who attributed the growth enhancement of Cyanobacteria* Aulosira fertilissima* that was grown in the presence of malathion to their capability to utilize this compound as sole sources of phosphorus in the absence of inorganic phosphate from the medium.

## 5. Conclusions

The present study is the first evidence of the ability of* A. oryzae*,* N. muscorum,* and* S. platensis* to biodegrade and utilize malathion as a source of phosphorus. Overall, the data obtained highlight the efficiency of algal strains to grow under high concentrations of malathion with enhancement of biomass carbohydrate and protein content. Moreover,* N. muscorum* overtopped the other strains in removing more than 90% of malathion. Hence, work in this regard should continue to characterise the genetic and enzymatic components responsible for the utilization of malathion and other organophosphorus pesticides of this strain in order to evaluate its efficiency for the bioremediation of these environmental pollutants.

## Figures and Tables

**Table 1:** The growth of algal strains, expressed as cell count (number of cells ×10^6^/mL), under unlimitation and P-limitation conditions with the addition of malathion.

Algal strains	*A. oryzae *	*N. muscorum *	*S. platensis *
Unlimitation	24.7 ± 2.9	6.6 ± 0.5	8.8 ± 0.1
P-limitation	1.2 ± 0.3^a^	1.4 ± 0.2^a^	4.2 ± 0.4^a^
P-limitation with different concentrations of malathion (ppm)			
0.02	4.4 ± 0.5^b^	2.1 ± 0.3^b^	6.4 ± 1.1^b^
0.2	5.4 ± 1.2^b^	4.3 ± 0.3^b^	7.0 ± 0.9^b^
2	5.6 ± 0.6^b^	4.8 ± 0.5^b^	7.2 ± 0.9^b^
20	13.4 ± 0.6^b^	4.9 ± 0.2^b^	7.8 ± 0.7^b^
50	22.6 ± 0.8^b^	7.1 ± 0.1^b^	8.2 ± 0.7^b^
100	26.1 ± 2.4^b^	7.6 ± 0.5^b^	0.0 ± 0.0^b^

Values are means of three replicates ± standard errors.

^a^Significant decrease compared with unlimitation condition.

^b^Significant increase compared with P-limitation condition.

**Table 2:** Total phosphorus content (mg/g dry weight) of algal biomass under unlimitation and P-limitation condition with the addition of malathion.

Algal strains	*A. oryzae *	*N. muscorum *	*S. platensis *
Unlimitation	10.1 ± 0.1	9.2 ± 0.1	19.8 ± 0.1
P-limitation	2.2 ± 0.1^a^	3.6 ± 0.1^a^	0.7 ± 0.1^a^
P-limitation with different concentrations of malathion (ppm)			
0.02	6.9 ± 0.0^b^	11.2 ± 0.0	1.0 ± 0.0
0.2	8.8 ± 0.1^b^	4.3 ± 0.1^b^	0.9 ± 0.1^b^
2	11.6 ± 0.1^b^	4.7 ± 0.1^b^	0.9 ± 0.1^b^
20	13.7 ± 0.1^b^	5.8 ± 0.1^b^	3.8 ± 0.1^b^
50	14.0 ± 0.1^b^	6.6 ± 0.1^b^	4.1 ± 0.1^b^
100	15.9 ± 0.1^b^	10.4 ± 0.1^b^	0.0 ± 0.0^b^

Values are means of three replicates ± standard errors.

^a^Significant decrease compared with unlimitation condition.

^b^Significant increase compared with P-limitation condition.
